# Polymorphisms of genes coding for insulin-like growth factor 1 and its major binding proteins, circulating levels of IGF-I and IGFBP-3 and breast cancer risk: results from the EPIC study

**DOI:** 10.1038/sj.bjc.6602936

**Published:** 2006-01-10

**Authors:** F Canzian, J D McKay, R J Cleveland, L Dossus, C Biessy, S Rinaldi, S Landi, C Boillot, S Monnier, V Chajès, F Clavel-Chapelon, B Téhard, J Chang-Claude, J Linseisen, P H Lahmann, T Pischon, D Trichopoulos, A Trichopoulou, D Zilis, D Palli, R Tumino, P Vineis, F Berrino, H B Bueno-de-Mesquita, C H van Gils, P H M Peeters, G Pera, E Ardanaz, M-D Chirlaque, J R Quirós, N Larrañaga, C Martínez-García, N E Allen, T J Key, S A Bingham, K-T Khaw, N Slimani, T Norat, E Riboli, R Kaaks

**Affiliations:** 1 International Agency for Research on Cancer, Lyon, France; 2 Institut Gustave Roussy, Villejuif, France; 3 German Cancer Research Centre, Heidelberg, Germany; 4 German Institute of Human Nutrition, Potsdam, Germany; 5 University of Athens Medical School, Athens, Greece; 6 CSPO-Scientific Institute of Tuscany, Florence, Italy; 7 Cancer Registry, Azienda Ospedaliera ‘Civile MP Arezzo’, Ragusa, Italy; 8 Imperial College, London, UK; 9 University of Torino, Turin, Italy; 10 National Cancer Institute, Milan, Italy; 11 National Institute of Public Health and the Environment, Bilthoven, The Netherlands; 12 Julius Center for Health Sciences and Primary Care, University Medical Center, Utrecht, The Netherlands; 13 Catalan Institute of Oncology, Barcelona, Spain; 14 Instituto de Salud Pública, SNS-O, Pamplona, Spain; 15 Epidemiology Department, Murcia Health Council, Spain; 16 Public Health Directorate, Consejería de Sanidad y Servicios Sociales de Asturias, Oviedo, Spain; 17 Public Health Division of Gipuzkoa, Health Department of the Basque Country, San Sebastián, Spain; 18 School of Public Health of Andalucia, Granada, Spain; 19 Cancer Research UK, Epidemiology Unit, University of Oxford, Oxford, UK; 20 MRC Dunn Human Nutrition Unit, Welcome Trust/MRC Building, Cambridge, UK; 21 Clinical Gerontology Unit, Addenbrooke's Hospital, Cambridge, UK

**Keywords:** IGF-I, IGFBP-3, IGFBP-1, IGFALS, single nucleotide polymorphisms, breast cancer

## Abstract

Insulin-like growth factor I (IGF-I) stimulates cell proliferation and can enhance the development of tumours in different organs. Epidemiological studies have shown that an elevated level of circulating IGF-I is associated with increased risk of breast cancer, as well as of other cancers. Most of circulating IGF-I is bound to an acid-labile subunit and to one of six insulin-like growth factor binding proteins (IGFBPs), among which the most important are IGFBP-3 and IGFBP-1. Polymorphisms of the *IGF1* gene and of genes encoding for the major IGF-I carriers may predict circulating levels of IGF-I and have an impact on cancer risk. We tested this hypothesis with a case–control study of 807 breast cancer patients and 1588 matched control subjects, nested within the European Prospective Investigation into Cancer and Nutrition. We genotyped 23 common single nucleotide polymorphisms in *IGF1*, *IGFBP1*, *IGFBP3* and *IGFALS*, and measured serum levels of IGF-I and IGFBP-3 in samples of cases and controls. We found a weak but significant association of polymorphisms at the 5′ end of the *IGF1* gene with breast cancer risk, particularly among women younger than 55 years, and a strong association of polymorphisms located in the 5′ end of *IGFBP3* with circulating levels of IGFBP-3, which confirms previous findings. Common genetic variation in these candidate genes does not play a major role in altering breast cancer risk in Caucasians.

Insulin-like growth factor-I (IGF-I) is a peptide growth factor that exerts mitogenic and metabolic activities that are regulators of growth, survival and cell differentiation in a number of cell and tissue types. A number of epidemiologic studies have shown a direct association between circulating IGF-I levels and breast cancer risk, especially in young women (Peyrat *et al*, 1993; Bruning *et al*, 1995; Bohlke *et al*, 1998; Hankinson *et al*, 1998; Toniolo *et al*, 2000; Muti *et al*, 2002; Yu *et al*, 2002). Elevated circulating IGF-I also has been found to be a potential cancer risk factor for cancers of other organs, such as the prostate (Mantzoros *et al*, 1997; Chan *et al*, 1998; Wolk *et al*, 1998; Stattin *et al*, 2000) and the colorectum (Ma *et al*, 1999; Giovannucci *et al*, 2000; Kaaks *et al*, 2000; Palmqvist *et al*, 2002).

Most of the circulating IGF-I originates from the liver. In the circulation, it is either free or bound to one of six high-affinity binding proteins, which regulate IGF-I activity. About 90% of the circulating IGF-I is bound to insulin-like binding protein-3 (IGFBP-3), which forms a ternary complex with an acid-labile subunit (ALS). The smaller complexes are able to pass the vascular endothelial barrier and therefore may be important modulators of IGF-I activity at the cellular level (Jones *et al*, 1993; Rajaram *et al*, 1997). There is physiologic evidence of a role for IGFBPs to work either in an IGF-I-dependent, such as increasing the half-life of IGF-I and modulating access to the IGF-I receptor, or IGF-I-independent fashion by mediating their effects directly on target cells, where they generally have a proapoptotic role (Jones *et al*, 1993; Perks *et al*, 1999; Gleeson *et al*, 2001; Mohan and Baylink, 2002).

While nutrition is an important determinant of circulating IGF-I levels (Thissen *et al*, 1994; Kaaks and Lukanova, 2001), heritability studies have shown that up to 40–60% of the variation in circulating IGF-I levels is determined by genetic factors (Harrela *et al*, 1996; Verhaeghe *et al*, 1996; Hong *et al*, 1997; Hall *et al*, 1999). So far, however, only few studies have been conducted to identify specific gene variants that influence circulating IGF-I levels, even though such research is currently intensifying (Hasegawa *et al*, 2000; Lopez-Bermejo *et al*, 2000; Jernstrom *et al*, 2001a; Vaessen *et al*, 2001).

For the present study, we focused on 23 common single nucleotide polymorphisms (SNPs) that we estimated would have the highest chance of having an impact on either the expression or function of IGF-I and of molecules (IGFBP-1, IGFBP-3 and IGFALS) involved in IGF-I transport. We conducted a case–control study of 807 breast cancer patients and 1588 matched control subjects, nested within the cohorts of the European Prospective Investigation into Cancer and Nutrition (EPIC) (Riboli *et al*, 2002; Bingham and Riboli, 2004), and examined relationships of these polymorphisms with circulating IGF-I and IGFBP-3 levels, as well as breast cancer risk.

## MATERIAL AND METHODS

### The EPIC study

The EPIC cohort consists of about 370 000 women and 150 000 men, aged 35–69 years, recruited between 1992 and 1998 in 23 research centres in 10 Western European countries. Although detailed information on ethnicity of EPIC subjects is not available, in practice, recruitment has not been conducted in large cosmopolitan urban areas; therefore, owing to the current ethnic composition of the regions involved in the study, we estimate that the vast majority (>97%) of subjects recruited in the EPIC cohort are of Caucasian origin. All EPIC study subjects provided anthropometric measurements (height, weight, waist and hip circumferences) and extensive, standardised questionnaire information about medical history, diet, physical activity, smoking and other lifestyle factors. Women also answered questions about menstrual and reproductive history, hysterectomy, ovariectomy and use of exogenous hormones for contraception or treatment of menopausal symptoms. In addition, about 240 000 women and 140 000 men provided a blood sample.

Cases of cancer occurring after recruitment into the cohort are identified through local and national cancer registries in seven of the 10 countries, and in France, Germany and Greece by a combination of contacts with national health insurances and/or active follow-up through the study subjects or their next of kin. Follow-up on vital status, to monitor the population remaining at risk for cancer, is achieved through record linkage with mortality registries. In all EPIC study centres, cancer diagnosis is confirmed through comprehensive review of pathology reports, and checks for completeness of follow-up are made periodically. A fully detailed description of the EPIC study has been published elsewhere (Riboli *et al*, 2002; Bingham and Riboli, 2004).

### Selection of case and control subjects

Cases and controls from the present study were from 16 of the 23 EPIC recruitment centres, in seven of the 10 countries participating in EPIC (UK, Germany, The Netherlands, France, Spain, Italy and Greece), and were part of a larger nested case–control study on serum hormones and breast cancer risk, reported in detail elsewhere (Kaaks *et al*, 2005a, 2005b; Rinaldi *et al*, manuscript submitted).

Case subjects were selected among women who developed breast cancer after their recruitment into the EPIC study, and before the end of the study period, for each study centre defined by the latest end-date of follow-up. Women who used any hormone replacement therapy at the time of blood donation, or any exogenous hormones for contraception or medical purposes, and who had previous diagnosis of cancer (except non-melanoma skin cancer) were excluded from the study, because each of these various factors could have altered circulating hormone levels.

For each case subject with breast cancer, two control subjects were chosen at random from among cohort members alive and free of cancer (except non-melanoma skin cancer) at the time of diagnosis of the index case. Control subjects were matched to the cases by study centre where the subjects were enrolled in the cohort, as well as by menopausal status (premenopausal, postmenopausal, perimenopausal/undefined), age (±6 months) at enrolment, follow-up time, fasting status, time of the day of blood donation and phase of the menstrual cycle for premenopausal women (assessed according to criteria defined by Kaaks *et al*).

Approval for the study was given by the relevant Ethical Committees, both at the IARC and in each of the EPIC recruitment centres.

### Identification and selection of SNPs

We collected data on polymorphisms from publicly available databases, such as dbSNP (http://www.ncbi.nlm.nih.gov/SNP/), SNPper (http://snpper.chip.org/) and Frequency Finder (http://bluegenes.bsd.uchicago. edu/frequencyfinder/). We complemented database searches with literature review and, for *IGFBP1* and *IGFBP3*, with analysis of 95 subjects from the EPIC population by denaturing high-performance liquid chromatography (DHPLC; Xiao and Oefner, 2001).

To be included in the study, polymorphisms had to be located in exons (including untranslated regions), exon–intron junctions or promoter regions of a gene of interest, or otherwise should be within intronic regions that showed greater than 80% homology between human and mouse (as reported by the UCSC Genome Browser, http://genome.ucsc.edu/), and thus were likely to harbour regulatory sequences. We included only polymorphisms whose existence in Caucasians is documented, either according to literature data or to our own experimental analysis by DHPLC. All new SNPs identified in our laboratory by DHPLC searches have been deposited in dbSNP (http://www.ncbi.nlm.nih.gov/SNP). Among all polymorphisms thus identified, we retained only those with a minor allele frequency ⩾5% in Caucasians or those that result in an amino-acid change and had a minor allele frequency ⩾1%. Finally, we particularly favoured the inclusion of all polymorphisms that were previously reported in the literature to be associated with cancer risk and/or levels of circulating hormones. In total, this strategy led to a list of 26 SNPs for genotyping.

### Genotyping

Buffy coat samples for the study subjects were retrieved from the EPIC biorepository and DNAs were extracted on an Autopure instrument (Gentra Systems, Minneapolis, MN, USA) with Puregene chemistry (Gentra Systems, Minneapolis, MN, USA).

Genotyping was performed by the 5′ nuclease assay (TaqMan). The order of DNAs from cases and controls was randomised on PCR plates in order to assure that an equal proportion of cases and controls could be analysed simultaneously. TaqMan probes were synthesised by either Applied Biosystems, Foster City, CA, USA (with MGB chemistry) or Proligo, Paris, France (with or without LNA chemistry). Sequences of primers and probes are reported in Supplementary Table 1. For one SNP, a genotyping assay could not be designed and for two more SNPs, TaqMan assays were generated but provided poor genotyping results. This left 23 polymorphisms that were genotyped on the DNAs of cases and controls (Table 1). The reaction mix included 10 ng genomic DNA, 5 pmol of each primer, 1 pmol of each probe and 2.5 *μ*l of 2 × master mix (Applied Biosystems) in a final volume of 5 *μ*l. The thermocycling included 50 cycles with 30 s at 95°C followed by 60 s at 60°C. PCR plates were read on an ABI PRISM 7900HT instrument (Applied Biosystems). Laboratory personnel was kept blinded to case–control status throughout the study. Genotyping call rates ranged between 95.27 and 99.44%. The distributions of genotypes of all polymorphisms were in agreement with the Hardy–Weinberg equilibrium (calculated in the control group). Repeated quality control genotypes (8% of the total) showed greater than 99% concordance for all assays.

### Hormone measurements

Measurements of IGF-I and IGFBP-3 were performed in the laboratory of the Hormones and Cancer, at IARC, using enzyme-linked immunosorbent assays from Diagnostic System Laboratories (DSL, Webster, TX, USA). The IGF-I assays included an acid–ethanol precipitation step to eliminate IGF-I binding proteins, to avoid their interference with the IGF-I measurement. Measurements were performed on never thawed serum sample aliquots. The mean intra- and inter-batch coefficients of variation were 6.2 and 16.2% respectively for IGF-I, and 7.2 and 9.7% respectively for IGFBP-3.

### Statistical analysis

Individual haplotype frequencies (i.e. estimated numbers of copies of haplotypes) were reconstructed using the program ‘tagSNPs’ (http://www-rcf.usc.edu/~stram/ tagSNPs.html; Stram *et al*, 2003a, 2003b). This program calculates, for each individual, the expected numbers of copies (‘dosages’) of each of the haplotypes compatible with the individuals' SNP genotypes. This method takes into account uncertainties in the haplotype reconstruction for individuals who are heterozygous for two or more of the SNPs within a given gene. Haplotype dosages are estimated from the individuals' SNP genotype data and from overall haplotype frequency estimates for the full study population (cases and controls combined) estimated by a maximum likelihood method. For each haplotype, the dosage values range from 0 to 2.0 (alleles), and for each individual these dosage values add up to a total value of 2.0 across all possible haplotypes.

All association analyses, at the level of individual SNPs or gene loci, were performed under different assumed modes of inheritance of effect – dominant, recessive or codominant – associated with alleles. In the ‘dominant’ model, circulating peptide levels or disease risks were compared between subjects carrying at least one copy of the rare allele and those who had none; in the ‘recessive’ model, the comparison was between those who were homozygous for the rare allele and all others; in the ‘codominant’ model, individuals’ peptide levels or the logarithm of disease risk were linearly related to the number of copies of an allele (0, 1 or 2 for SNP alleles, or dosages for the haplotype) carried by the individuals. For rare alleles, with a frequency less than 20% (i.e. a prevalence of homozygous recessive allele carriers less than 4%), only the dominant model was used. To test whether any association of gene variants with breast cancer risk could be mediated by alterations in circulating levels of IGF-I and/or IGFBP-3, these associations were also estimated with adjustment for serum peptide levels.

Relationships of polymorphic gene variants with serum levels of IGF-I and IGFBP-3 were estimated by standard normal regression models, stratified by EPIC recruitment centre and further adjusted for age. Relationships of polymorphic variants with breast cancer risk (odds ratios) were estimated using conditional logistic regression models, applied on the matched case–control sets. Both series of analyses were performed at the level of single SNP loci, as well as at the level of haplotypes (using the haplotype dosage values). Haplotype analyses were performed at the level of full gene loci – that is, including haplotypes based on all of the SNPs in that gene – and for the *IGF1* and *IGFBP3* genes also at the level of well-delineated haplotype blocks within a gene. In all haplotype analyses, the most common haplotype was used as the reference category.

Adjustment for potentially relevant variables (body mass index, adult height, total caloric intake) did not alter significantly the results (data not shown). Exclusion of cases diagnosed within a year of blood collection also did not have an impact on the results (data not shown).

Reconstruction of haplotype blocks within each gene was performed with Haploview (http://www.broad.mit.edu/perso nal/jcbarret/haploview; Barrett *et al*, 2005). Block boundaries were determined using the criterion of Gabriel *et al* (2002).

Subgroup analyses on women with a breast cancer diagnosis either before (45% of the subjects) or after age at diagnosis of 55 years (the age at which over 99% of women enrolled in the EPIC cohort declared themselves menopausal) were used to examine whether associations of gene variants with breast cancer risk differed between women with cancer at approximately premenopausal or postmenopausal age, and possible heterogeneity of effect between these two age groups was tested using a χ^2^ test.

We estimated the false positive reporting probability (FPRP) for statistically significant observations based on the methods described by Wacholder *et al* (2004). Prior probability is likely to be influenced by the biological knowledge of the gene, the functional significance of the variants and the available epidemiological evidence. It remains a subjective measure that may vary from one investigator to another based on the importance they assign to the different pieces of evidence. For this reason, we have calculated FPRP for a range of prior probabilities from 50 to 0.1%. We considered that a prior probability of 50% might be acceptable when there was a very strong biological plausibility with consistent epidemiological evidence (i.e. the association between SNPs in the 5′ part of *IGFBP3* and IGFBP-3 level), and a prior probability of 0.1% may be appropriate when the biological knowledge and epidemiological data were both inadequate (i.e. the majority of other SNPs, whose exact function is not known, and epidemiological data do not exist).

## RESULTS

A total of 807 incident cases of breast cancer from the EPIC cohort and 1588 matched controls were included in our study. The mean age of study subjects at blood donation was 55 years (5th–95th percentile: 39.9–68.7 years). For cases, the mean age at diagnosis was 57 years (5th–95th percentile: 42–71 years). Based on the questionnaire data, 32% of the subjects were premenopausal at blood donation, 10% were perimenopausal or of unknown menopausal status and 58% were postmenopausal. Cases had a significantly lower number of full-term pregnancies than controls (means: 2.35 *vs* 2.47, *P*=0.02) and were significantly older at first full-term pregnancy (26 *vs* 25.5 years in controls, *P*=0.02). Age at menarche did not differ between cases and controls, nor did body mass index. Serum levels of IGF-I adjusted for age and centre were not significantly different between cases and controls (means: 248.7 *vs* 244.4 ng ml^−1^, *P*=0.15), nor for the subgroups subjects with cancer diagnosis before or at the age of 55 years (272.0 *vs* 270.7; *P*=0.79), or after (224.0 *vs* 217.5; *P*=0.08). Case subjects did show higher mean levels of serum IGFBP-3 than controls (means: 3422 *vs* 3361 ng ml^−1^, *P*=0.04). The latter difference, however, was due mostly to the subgroup with cancer diagnosis after age 55 years (3473 *vs* 3378; *P*=0.02), and was not clearly visible among the younger women (3190 *vs* 3173; *P*=0.66). Details on the relationships of IGF-I and IGFBP-3 with breast cancer risk, with an extended series of 1195 beast cancer cases and 2321 control subjects, will be reported elsewhere (Rinaldi *et al*, manuscript submitted).

The number of SNPs typed per gene ranged from three for *IGFALS* to eight for *IGFBP3*. Results of associations between individual SNPs and cancer risk and circulating IGF-I and IGFBP-3 levels are reported in Table 2. Supplementary Tables 2a–f report results of analyses relating hormone levels and breast cancer risk to haplotypes.

**Figure 1:**
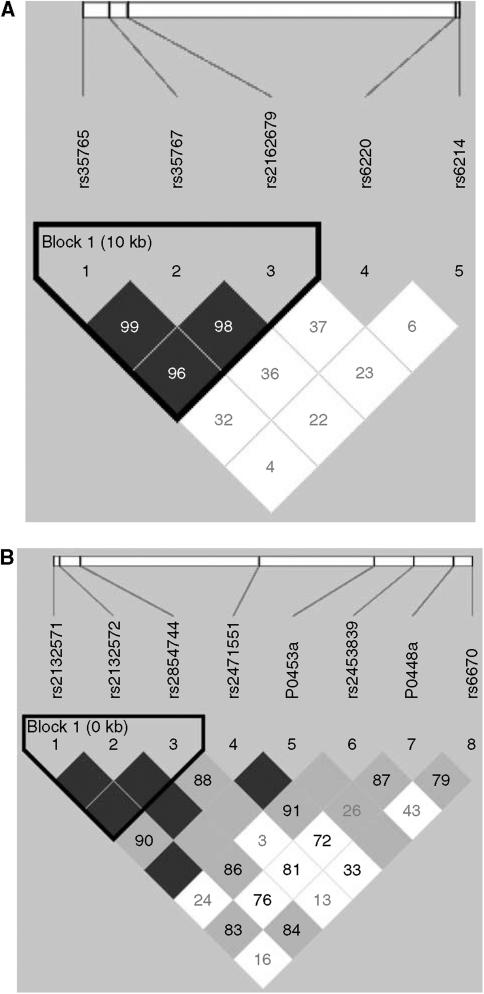
(**A**) Graphical representation of LD and block structure of *IGF1*. (**B**) Graphical representation of LD and block structure of *IGFBP3*. The upper bars represent SNPs and physical distances among them. Numbers within squares are pairwise *D*′ values. Absence of value means *D*′=1. The colour code shows confidence boundaries of LD estimations: black shows evidence of LD, white shows evidence of recombination and grey shows uninformative pairs. Linkage disequilibrium blocks were defined according to the algorithm of Gabriel *et al* (2002). Internal references are used for polymorphisms not present in dbSNP.

In the *IGF1* gene, we noted an association of the rs2162679 SNP, with the G/G genotype being associated with a reduction in risk of breast cancer (*P*=0.05) and also a modest effect in the heterozygote (*P*-trend for codominant model=0.03). Interestingly, when the cases were stratified by age of onset (less than 55 years old or greater than or equal to 55 years old), this reduction appears to be confined to breast cancer cases with an early age of diagnosis, at 55 years or before, and was strongest (odds ratio 0.17, confidence interval 0.07–0.56) in the homozygous G/G (*P*=0.005). This association was also found to be statistically significant under the dominant (*P*=0.01) and codominant (*P*=0.002) models. Linkage disequilibrium (LD) reconstruction of this region shows that there is strong LD between the rs2162679 and the two surrounding SNPs, rs35765 and rs35767. As expected, rs35767 and rs35765 also showed a reduction in breast cancer risk among the entire set, although the only statistically significant result was seen for rs35767 when testing for trend (*P*=0.04). However, as with rs2162679, the reduction effect was statistically significant in both rs35767 and rs35765 for the breast cancer cases diagnosed at age 55 years or before. Haplotype analysis of the *IGF1* data showed only an association between one rare haplotype and cancer risk (Supplementary Table 2). When we analysed the block structure of *IGF1*, based on our control genotypes, we noted a clear haplotype block that contains rs35765, rs35767 and rs2162679 (Figure 1A). When we restricted our haplotype analysis to only this block, we observed an association between cancer risk and haplotype hCTG, which has a frequency of 4% and includes the rare alleles of rs35767 and rs2162679. This association was not stronger than that seen with the individual SNPs (Supplementary Table 2).

We also noted a significant decrease in circulating IGFBP-3 levels with the rare homozygous states for both rs2162679 and rs35767 (again representing the LD between these markers). In contrast to the association of breast cancer risk, with the same markers, this effect appeared restricted to a later age of onset in our age-stratified analysis. When we performed the same analysis by using the haplotype block that spans the 5′ end of the gene and includes rs2162679 and rs35767, the same haplotype that is associated with reduced cancer risk appeared also to be strongly associated with decreased IGFBP-3 levels (*P*<0.0001). The only other result of interest in the *IGF1* gene was a relatively modest dominant effect of polymorphisms rs35765, rs35767 and rs6220, resulting in a higher mean circulating IGF-I level, and visible also in the haplotype analysis.

For the *IGFBP3* gene, we observed that a number of previous studies showed an increase in circulating IGFBP-3 levels associated with the A allele of a polymorphism in the promoter of *IGFBP3* at position −202 (rs2854744) (*P*⩽0.0001). Reflecting the strong amount of LD in this area (Figure 1B), we also noted a strong association between increased IGFBP-3 levels and alleles of the surrounding polymorphisms, rs2132571, rs2132572, rs2471551, which belong to the same haplotype block (Supplementary Table 2).

The polymorphism P0453 of *IGFBP3* also showed a slight increase in the mean circulating IGF-I protein levels and one *IGFBP3* haplotype showed an increase in risk of breast cancer, under a recessive model.

Polymorphic variations in the *IGFBP1* gene did not show associations with any of the end points. Homozygous carriers of the polymorphism rs3751893 in *IGFALS*, however, showed a significant reduction in mean circulating IGF-I levels.

## DISCUSSION

We have performed a large-scale association study, nested in the EPIC cohort, to assess the role of genetic variation of *IGF1* and of the genes encoding the major IGF-I binding proteins on risk of breast cancer and on circulating levels of IGF-I and IGFBP-3. We genotyped 23 SNPs in the four candidate genes. Our criteria for selecting the SNPs to study were proven existence in the Caucasian population, high allele frequency and/or high chance of having an impact on gene expression or function of the gene product. Our selection of SNPs to be typed was not based on a formal haplotype tagging approach, because we estimated that the available information (as of the time of writing this report) was insufficient to do so accurately. In view of the relative paucity of publicly available (e.g. HapMap) data, an accurate haplotype tagging approach would have required complete resequencing of the gene region in a sufficient number of subjects, in order to establish a complete catalogue of polymorphisms and to examine LD patterns between them. Such effort was beyond the scope of the present project. Nevertheless, we feel that it is unlikely that many new common polymorphisms, which were the focus of our investigation, would have been discovered by systematic resequencing.

The use of a multicentric study raises the possibility of confounding by population stratification. Although 97% of EPIC subjects are estimated to be of Caucasian origin, there could still be confounding by population stratification if SNP or haplotype allele frequencies varied between the subject recruitment areas, while at the same time there was also variation in average breast cancer incidence rates. We did not observe wide differences in allele frequencies between different countries in our study (data not shown). In addition, breast cancer cases and control subjects were systematically matched for the study centre where they had been recruited into the EPIC cohorts, and we adjusted all statistical analyses of association between SNPs, hormone levels and breast cancer risk for the factor ‘recruitment centre’. We believe therefore that confounding by population stratification is not an issue in our study.

We have found an association with decreased breast cancer risk of a haplotype located in the 5′ part of *IGF1*. This association was particularly strong among women younger than 55 years.

No other variant among the ones we studied showed any association with cancer risk, except for two haplotypes of *IGFBP3*. These associations are likely to be chance findings, as they are based on small numbers.

Our results suggest that in none of the four genes we examined were there any SNPs that had a strong impact on circulating levels of IGF-I.

Our results are in conflict with a previous study (Ukkola *et al*, 2001), which found that carriers of the less frequent G variant allele of SNP rs3793344, located in a region of intron 1 of *IGFBP1*, which harbours sequences affecting gene expression, had significantly lower circulating levels of IGF-I both before and after overfeeding. Subjects carrying two copies of the A allele had lower IGF-I concentrations before overfeeding, which were further decreased after overfeeding compared to subjects carrying the rarer G allele. This may indicate that the AA genotype results in higher IGFBP-1 concentrations, which could decrease available IGF-I. We found no evidence of higher IGF-I concentrations with this polymorphism. It is possible that the previously reported association (Ukkola *et al*, 2001) is linked to the peculiar study conditions (long-term caloric surplus). Alternatively, the association could be due to a statistical fluctuation caused by the small sample size of the study (12 pairs of monozygotic twins).

The finding supported by the strongest statistical evidence in our study is the association of polymorphisms in the 5′ region of *IGFBP3* with circulating levels of IGFBP-3. This association has been reported in several previous studies, and has been ascribed to a polymorphism located at position −202 (rs2854744) from the transcription start site (Deal *et al*, 2001; Jernstrom *et al*, 2001b; Schernhammer *et al*, 2003). In accordance with these previous reports, we also found a dose-dependent association of the A allele with increased levels of circulating IGFBP-3. In addition, we observed strong associations of all surrounding polymorphisms with IGFBP-3 levels (Supplementary Table 2). This leads to the question which of these various polymorphisms would be the functional polymorphism causing the association with IGFBP-3 levels. In a recent study *in vitro*, a construct including only the A allele of the rs2854744 SNP variant was found to increase promoter activity, suggesting that rs2854744 is the functional variant that affects IGFBP-3 transcription, and that the associations we have observed with the other polymorphisms of *IGFBP3* are exclusively due to strong LD (Deal *et al*, 2001).

The −202 polymorphism also has been studied in relation to risk of breast cancer (Deal *et al*, 2001; Schernhammer *et al*, 2003) and other cancers (Wang *et al*, 2003; Li *et al*, 2004; Slattery *et al*, 2004). In agreement with most previous studies, we noted no association between this allele in our series as a whole, or in our stratified analysis based on age of onset. Probably, the effect of this polymorphism on circulating IFGBP-3 levels (estimated in 6–9% of variation) (Jernstrom *et al*, 2001b; Schernhammer *et al*, 2003) is not sufficient, by itself, to alter amounts of bioavailable IGF-I sufficiently to lead to a substantial change in cancer risk. In one recent study, however, the C allele of the −202 polymorphism was found to be associated with more advanced disease status in prostate cancer, even though it was not associated with prostate cancer risk overall (Wang *et al*, 2003).

Besides polymorphic variation in the *IGFBP3* gene, another strong predictor of variability in IGFBP-3 levels was the same *IGF1* haplotype that was associated, in the same direction, with cancer risk. This was particularly evident when the analysis was restricted to the haplotypes observed within the strong LD block in the 5′ region of the gene. It is difficult to interpret these associations, with either IGFBP-3 levels or cancer risk, as for none of these SNPs is there any evidence to suggest a possible functional role. Little appears to be known about what genomic elements are involved in the regulation of *IGF1* transcription, except for a region immediately upstream of the transcription start (Porcu *et al*, 1994; Rubini *et al*, 1994), which however harbours no known genetic variation. We have not typed a CA repeat polymorphism located in the 5′ region of *IGF1*, between SNPs rs35767 and rs2162679, which has been frequently included in previous studies. Several epidemiological studies on the association between this polymorphism and breast cancer risk or IGF-I levels (Jernstrom *et al*, 2001a; Yu *et al*, 2001; Figer *et al*, 2002; Missmer *et al*, 2002; DeLellis *et al*, 2003) have yielded highly conflicting results. This microsatellite polymorphism has a large number of alleles, which results in fractioning the study population into a large number of classes. Even with a large sample size, this makes statistical analysis very difficult. Moreover, there are no studies that show a functional role for this microsatellite. We believe that it is unlikely that the *IGF1* CA repeat has a functional role of its own, and that associations previously reported with this microsatellite are likely to reflect LD with the same SNPs that we included in our study or with other, yet unknown SNPs located in the region. In any case, the level of LD in the 5′ region of the gene is so high that an association study approach alone will not be able to single out one particular variant as that causing variation in IGFBP-3 levels and, possibly, cancer risk. Moreover, genetic variation in the promoter of *IGF1* should have an impact on circulating IGFBP-3 only through a modification of circulating IGF-I, which we observe only at modest levels. Functional studies will be needed to clarify this point.

Other polymorphic variants that were found to be associated with IGFBP-3 levels – a haplotype in the 5′ region of *IGF1*, in homozygosity, and SNP rs3751893 of *IGFALS* – are difficult to evaluate, because of the small number of subjects carrying the alleles that showed the associations.

In summary, the main findings of our study were thus a weak but nominally significant association of a block of polymorphisms located at the 5′ end of the *IGF1* gene with breast cancer risk, particularly among women younger than 55 years, and an association of polymorphisms located in the 5′ region of *IGFBP3* with circulating levels of IGFBP-3. The large number of statistical tests we have performed raises the issue of potential false positives. An alternative to applying a Bonferroni's correction, which is generally too conservative because of statistical dependence between tests for multiple SNPs that are in LD, is the use of a Bayesian approach, such as the recently introduced calculation of FPRP (Wacholder *et al*, 2004). Given the absence of previous functional or epidemiologic data on the *IGF1* SNPs we found associated with breast cancer risk, we calculated FPRPs by using a prior probability of true association. Calculated FPRPs were high even with a relatively high prior probability of 0.01 that variants would have an association with breast cancer risk or with circulating peptide levels. Our finding relating *IGFBP3* SNPs with serum IGFBP-3 levels, however, is strongly backed by previous epidemiologic and functional findings and is also supported by very low *P*-values in our study, and resulted in very low FPRPs over a wide range of prior probabilities of true association.

In conclusion, our results show a number of genetic variants associated with circulating hormone levels, including a convincing association of *IGFBP3* SNPs with IGFBP-3 levels. On the other hand, we have found only weak or no associations of genetic variants in *IGF1*, *IGFBP1*, *IGFBP3* and *IGFALS* with breast cancer risk, and further large studies will be required to confirm our findings.

## Figures and Tables

**Table 1:** Polymorphisms used in the present study

**Polymorphism^a^**	**Alleles (major>minor)**	**Position in gene**	**Codon**
*IGF1*			
rs35765	C>A	Promoter region	
rs35767	C>T	Promoter region	
rs2162679	A>G	Intron 1	
rs6220	T>C	Exon 4; 3′ UTR	
rs6214	C>T	Exon 4; 3′ UTR	

*IGFBP1*			
rs1995051	G>A	Promoter region	
rs1065780	G>A	Promoter region	
rs9658194	C>A	Intron 1	
rs3828998	T>C	Intron 1	
rs3793344	A>G	Intron 1	
rs4988515	C>T	Exon 4	Cys230Cys
rs4619	A>G	Exon 4	Met253Ile

*IGFBP3*			
rs2132571	G>A	Promoter region	
rs2132572	G>A	Promoter region	
rs2854744	C>A	Promoter region	
rs2471551	G>C	Intron 1	
P0453^b^	C>T	Exon 4	Thr277Ile
rs2453839	A>G	Exon4; 3′ UTR	
P0448^b^	T>C	Exon4; 3′ UTR	
rs6670	A>T	Exon4; 3′ UTR	

*IGFALS*			
rs3751893	T>C	Exon 2	Asp70Asp
rs17559	C>T	Exon 2	Tyr462Tyr
rs2230053	G>A	Exon 2	Thr522Thr

UTR=untranslated region.

a Polymorphisms are identified by their dbSNP accession number.

b Internal references are used for polymorphisms not present in dbSNP.

**Table 2:** Associations between SNPs and breast cancer risk and mean IGF-I and IGFBP-3 levels adjusted for age and centre

			**Genotype**					
**Gene**	**SNP**		**Homozygous major**	**Heterozygous**	**Homozygous minor**	***P*_Codominant_ a**	***P*_Dominant_ b**	***P*_Recessive_ c**
*IGF1*	rs35765	Cases/controls	609/1148	169/362	17/37			
		OR (95% CI)	1.00	0.88 (0.72–1.08)	0.86 (0.48–1.54)	0.22	0.21	0.70
		Mean IGF-I	244.2	253.6	234.4	0.10	**0.02**	0.21
		Mean IGFBP-3	3379	3412	3371	0.46	0.36	0.87

	rs35767	Cases/controls	549/1016	201/432	22/62			
		OR (95% CI)	1.00	0.86 (0.71–1.05)	0.66 (0.40–1.09)	**0.04**	0.07	0.15
		Mean IGF-I	243.2	252.0	238.0	0.10	**0.02**	0.32
		Mean IGFBP-3	3377	3385	3204	0.25	0.66	**0.02**

	rs2162679	Cases/controls	570/1060	212/446	19/61			
		OR (95% CI)	1.00	0.88 (0.72–1.06)	0.57 (0.34–0.97)	**0.03**	0.07	0.05
		Mean IGF-I	244.7	251.5	239.3	0.23	0.08	0.35
		Mean IGFBP-3	3397	3382	3165	**0.04**	0.20	**0.003**

	rs6220	Cases/controls	405/813	325/592	59/126			
		OR (95% CI)	1.00	1.11 (0.92–1.33)	0.95 (0.68–1.32)	0.69	0.39	0.54
		Mean IGF-I	247.6	255.2	247.0	0.17	**0.03**	0.46
		Mean IGFBP-3	3420	3395	3363	0.22	0.26	0.38

	rs6214	Cases/controls	282/503	366/753	131/271			
		OR (95% CI)	1.00	0.88 (0.72–1.06)	0.88 (0.68–1.13)	0.22	0.15	0.64
		Mean IGF-I	246.7	244.3	244.9	0.56	0.45	0.91
		Mean IGFBP-3	3395	3344	3376	0.41	0.15	0.79

*IGFALS*	rs3751893	Cases/controls	546/1085	231/436	21/42			
		OR (95% CI)	1.00	1.06 (0.88–1.28)	0.98 (0.57–1.67)	0.67	0.59	0.89
		Mean IGF-I	251.4	249.8	226.4	0.06	0.23	**0.005**
		Mean IGFBP-3	3420	3375	3305	0.07	0.10	0.24

	rs17559	Cases/controls	620/1246	149/256	5/10			
		OR (95% CI)	1.00	1.17 (0.94–1.46)	1.02 (0.35–2.99)	0.19	0.17	1.00
		Mean IGF-I	244.9	248.5	227.9	0.60	0.45	0.32
		Mean IGFBP-3	3375	3344	3439	0.53	0.45	0.69

	rs2230053	Cases/controls	788/1551	13/16	0/1			
		OR (95% CI)	1.00	1.64 (0.75–0.36)	—	0.37	0.28	—
		Mean IGF-I	246.2	248.3	240.1	0.90	0.89	0.93
		Mean IGFBP-3	3385	3487	3751	0.35	0.38	0.59

*IGFBP1*	rs1995051	Cases/controls	413/766	302/627	60/110			
		OR (95% CI)	1.00	0.89 (0.74–1.07)	1.02 (0.73–1.42)	0.50	0.28	0.67
		Mean IGF-I	253.4	256.3	247.1	0.89	0.59	0.17
		Mean IGFBP-3	3239	3243	3261	0.74	0.82	0.71

	rs1065780	Cases/controls	304/555	356/741	114/207			
		OR (95% CI)	1.00	0.88 (0.73–1.07)	1.02 (0.78–1.33)	0.71	0.31	0.50
		Mean IGF-I	252.9	254.3	255.7	0.52	0.57	0.63
		Mean IGFBP-3	3234	3260	3210	0.91	0.61	0.35

	rs9658194	Cases/controls	291/521	253/498	25/51			
		OR (95% CI)	1.00	0.98 (0.81–1.19)	0.95 (0.59–1.55)	0.81	0.83	0.87
		Mean IGF-I	253.5	254.2	253.1	0.88	0.84	0.94
		Mean IGFBP-3	3222	3281	3264	0.08	0.05	0.78

	rs3828998	Cases/controls	291/521	332/686	110/194			
		OR (95% CI)	1.00	0.87 (0.71–1.05)	1.03 (0.78–1.35)	0.71	0.27	0.42
		Mean IGF-I	253.7	253.6	255.6	0.75	0.91	0.64
		Mean IGFBP-3	3198	3214	3183	0.93	0.76	0.56

	rs3793344	Cases/controls	305/549	350/742	115/203			
		OR (95% CI)	1.00	0.85 (0.70–1.02)	1.03 (0.79–1.35)	0.66	0.19	0.34
		Mean IGF-I	254.0	254.1	255.3	0.82	0.91	0.77
		Mean IGFBP-3	3233	3256	3212	0.93	0.64	0.41

	rs4988515	Cases/controls	715/1412	62/111	2/6			
		OR (95% CI)	1.00	1.11 (0.80–1.54)	0.67 (0.13–3.30)	0.72	0.62	0.62
		Mean IGF-I	253.9	258.6	239.9	0.57	0.46	0.56
		Mean IGFBP-3	3164	3213	3479	0.18	0.25	0.20

	rs4619	Cases/controls	347/665	347/698	89/175			
		OR (95% CI)	1.00	0.95 (0.79–1.15)	0.97 (0.73–1.30)	0.71	0.62	1.00
		Mean IGF-I	254.2	253.4	257.4	0.70	0.99	0.43
		Mean IGFBP-3	3174	3171	3124	0.41	0.67	0.28

*IGFBP3*	rs2132571	Cases/controls	393/714	308/674	75/135			
		OR (95% CI)	1.00	0.83 (0.69–0.99)	1.01 (0.74–1.38)	0.29	0.08	0.50
		Mean IGF-I	253.5	253.0	256.8	0.73	0.96	0.49
		Mean IFGBP-3	3322	3208	3034	**<0.0001**	**<0.0001**	**<0.0001**

	rs2132572	Cases/controls	501/980	234/478	42/59			
		OR (95% CI)	1.00	0.96 (0.80–1.17)	1.42 (0.94–2.15)	0.47	0.90	0.08
		Mean IGF-I	254.2	251.1	274.5	0.30	0.94	**0.002**
		Mean IFGBP-3	3266	3195	3206	**0.03**	**0.02**	0.61

	rs2854744	Cases/controls	208/407	368/743	200/369			
		OR (95% CI)	1.00	0.97 (0.79–1.20)	1.08 (0.85–1.38)	0.52	0.92	0.34
		Mean IGF-I	259.8	248.8	254.8	0.19	**0.01**	0.51
		Mean IFGBP-3	3101	3246	3389	**<0.0001**	**<0.0001**	**<0.0001**

	rs2471551	Cases/controls	485/928	248/508	38/59			
		OR (95% CI)	1.00	0.92 (0.76–1.12)	1.22 (0.80–1.86)	0.97	0.62	0.29
		Mean IGF-I	252.4	254.0	257.8	0.42	0.50	0.50
		Mean IFGBP-3	3285	3200	2994	**<0.0001**	**0.0002**	**0.0002**

	P0453^d^	Cases/controls	770/1506	10/29	—			
		OR (95% CI)	1.00	0.68 (0.33–1.39)	—	0.29	0.29	—
		Mean IGF-I	253.4	289.5	—	**0.001**	**0.001**	—
		Mean IFGBP-3	3243	3078	—	0.13	0.13	—

	rs2453839	Cases/controls	523/985	236/502	27/59			
		OR (95% CI)	1.00	0.89 (0.74–1.08)	0.87 (0.55–1.39)	0.22	0.20	0.67
		Mean IGF-I	254.1	253.2	255.0	0.90	0.83	0.87
		Mean IFGBP-3	3243	3232	3273	0.96	0.81	0.66

	P0448^d^	Cases/controls	566/1116	202/384	14/26			
		OR (95% CI)	1.00	1.05 (0.86–1.29)	1.07 (0.56–2.06)	0.61	0.61	0.87
		Mean IGF-I	255.0	250.5	245.9	0.13	0.15	0.47
		Mean IFGBP-3	3233	3272	3270	0.24	0.22	0.80

	rs6670	Cases/controls	445/887	287/572	43/61			
		OR (95% CI)	1.00	1.00 (0.83–1.19)	1.44 (0.95–2.18)	0.35	0.69	0.08
		Mean IGF-I	260.7	256.4	254.3	0.12	0.12	0.50
		Mean IFGBP-3	3263	3256	3368	0.48	0.87	0.11

a *P*-value for codominant model (trend).

b *P*-value for dominant model.

c *P*-value for recessive model.

SNP=single nucleotide polymorphism; IGF-I=insulin-like growth factor I; IGFBP-3=insulin-like binding protein-3; OR=odds ratio; CI=confidence interval.

Mean IGF-I and mean IFGBP-3 are means of hormone levels, expressed in ng/ml, for subjects (cases+controls) belonging to each genotype category, adjusted for age and centre.

d Internal references are used for polymorphisms not present in dbSNP.

*P*-values that reached statistical significance at the 0.05 level are reported in bold.
