# Supplementary material for: Polymorphisms of genes coding for insulin-like growth factor 1 and its major binding proteins, circulating levels of IGF-I and IGFBP-3 and breast cancer risk: results from the EPIC study
Source: Br J Cancer. 2006 Jan 10;94(2):299–307. doi: 10.1038/sj.bjc.6602936 (PMC2361124; doi:10.1038/sj.bjc.6602936)
Supplement: Supplementary Tables [file 94-6602936x1.doc]

**Supplementary Table 1.** PCR primers, probes and labels for TaqMan genotyping assays.

| Polymorphism^a^ | Primers (5’-3’) | Probes (5’-3’)^b^ |
| --- | --- | --- |
| ***IGF1*** |  |  |
| rs35765 | CATCTTCATACAATATTAATGACACA  TCCAATTCTAAGTTTTCTTACTCATT | HEX-LNA-CTT+CTCT+TTGG+CAA+TGATC  FAM-LNA-CTT+CTCTTT+GG+AA+AT+GATC |
| rs35767 | ABI assay-on-demand C____799146_10^c^ | ABI assay-on-demand C____799146_10^c^ |
| rs2162679 | TCTTCCAGTCCCAGTGTTTTGG  GAGGAGGTAAAAGCTCAACTGAACA | VIC-MGB-ATCTTCCACCCTCTACATAG  FAM-MGB-TTCCACCCCCTACATAG |
| rs6220 | AACAAAGAGATTTCTACCAGTGAAAGG  GCCTAGAAAAGAAGGAATCATTGTG | VIC-MGB-AGTAAAACCTCGTTTAATA  FAM-MGB-AGTAAAACCTTGTTTAATAC |
| rs6214 | TCTCAACAAAACTTTATAGGCAGTCTTC  GTGAAGGAAATAAGTCATAGACACTCTTAGAA | VIC-MGB-TGCAGACTTAACATGTT  FAM-MGB-AGTCATGCGGAAAA |
|  |  |  |
| ***IGFBP1*** |  |  |
| rs1995051 | GTTCCCAGAAATGGCTGTGTGA  GTCCATCCGTCCATCCTGTGT | HEX-MGB-CACATCATCGGCTGTGACTCCAACCTGC  FAM-MGB-CACATCATCGGCTGTAACTCCAACCTGC |
| rs1065780 | CCCCATCTCGCCTTTCCT  GCAAAAATTTAAAAATCCTGGGTAAC | VIC-MGB-TCTCAAATGCAGAAAA  FAM-MGB-CGTGAGAGGATTGAG |
| rs9658194 | GGGAAGGAGCTTGGGTCACC  TCCTTGAGTCTCCACTAAGCTATGT | HEX-AGCCCGCTCATTGAACGGTCTTGGCA  FAM-AGCCCGCTCATTGCACGGTCTTGGCA |
| rs3828998 | CCCGCTCATTGCACGGTCTT  GTCTCCACTAAGCTATGTGTGCC | HEX-LNA-CTCC+CAG+AG+CACGTC  FAM-LNA-CCC+AG+GGC+ACGT |
| rs3793344 | TGGAGACTCAAGGAGGAAGCTC  GTGCTGGCAAGGAGACTGGT | HEX-CCTCGTAGCCCAGGGATCTTTAGAGACCC  FAM-CCTCGTAGCCCAGAGATCTTTAGAGACCC |
| rs4988515 | GTCTTTGCAGTGTGAGACATCCAT  GGAGACCCAGGGATCCTCTTC | VIC-MGB-TGGTGCGTCTACC  FAM-MGB-TGGTGTGTCTACCC |
| rs4619 | CCCTGGGTCTCCAGAGATCAG  TTTCACGTGACAGAACATTATTTCATC | VIC-MGB-TGCCAGATGTATTTTA  FAM-MGB-CTGCCAGATATATTT |
|  |  |  |
| ***IGFBP3*** |  |  |
| rs2132571 | GTTAATTACGTTTCAGCAGTGC  TTAAGGCAGGGCTTTTCAAAT | HEX-LNA-TTT+TGTC+GTGGG+TG+TAA  FAM-LNA-TCT+TT+TGTC+ATGGG+TG+TAA |
| rs2132572 | TTAAGGACGCATTCGCTTGC  GCCCACAAATACGCATCTGA | HEX-LNA- CTTAAT+TGG+GGACT+TGCG  FAM-LNA-CTT+AAT+TGG+AGA+CTTGCGG |
| rs2854744 | CACCTTGGTTCTTGTAGACGACAA  GGCGTGCAGCTCGAGACT | VIC-MGB-TCCTCGTGCGCACG  FAM-MGB-CTCGTGCTCACGCC |
| rs2471551 | ACCTGGTTGCAACGTTAAGATTTT  CCGACTCACTAGCATTTCCTTAAAA | VIC-MGB-TTGTCTCTCTTGGGCG  FAM-MGB-TGTCTGTCTTGGGCG |
| P0453^d^ | CTGCTGGTGTGTGGATAAGTATGG  GTAGCAGTGCACGTCCTCCTT | VIC-MGB-CCTTGGTGGTGTAGC  FAM-MGB-CCCTTGATGGTGTAGC |
| rs2453839 | GCATCTGGTACTCGTTGCTTTGT  CCTGCAGGCTAATGGCACTAG | VIC-MGB-CTCAACTCATGTTTTC  FAM-MGB-CAACTCACGTTTTCA |
| P0448^d^ | GGCCCAGGATGGCTTTTG  ATTACTTGTGATGCCTCTGAATGTG | VIC-MGB-AGAGACAGGGAGAGTC  FAM-MGB-AGAGACGGGAGAGTC |
| rs6670 | CTATACTAGATAATCCTAGATGAAATGT  GCCAGACCTTCTTGGGTT | HEX-LNA-ATG+CTAT+TT+GATA+CAA+CTGT  FAM-LNA-TGC+TAT+AT+GATA+CAA+CTGT |
|  |  |  |
| ***IGFALS*** |  |  |
| rs3751893 | CTGCAGCTCCAGGAACCT  GCCACAGGGCTTGGGT | VIC-MGB-CCCGGGACTCCGTCAG  FAM-MGB-CCGGGACTCCATCAG |
| rs17559 | CTCCAACCAGCTCACGC  GAGACGTCCAGCCAGAA | HEX-LNA-AAGC+TG+GAG+TA+TC+TGC  FAM-LNA-AAGC+TGG+AGTA+CC+TGC |
| rs2230053 | CAGCCTCAGGAACAACTCACT  GCCACAGGCGCTCCA | VIC-MGB-CCTTCACACCGCAGCC  FAM-MGB-TCACGCCGCAGCC |
|  |  |  |

^a^ Polymorphisms are identified by their dbSNP accession number.

^b^ Fluorescent dye and presence of stabilizing molecules (MGB or LNA) are indicated. For LNA probes, a plus sign indicates bases modified with an LNA molecule

^c^ No information on sequences of primers and probes is available for assays purchased as predesigned Assays-on-demand from Applied Biosystems

^d^ Internal references are used for polymorphisms not present in dbSNP

**Supplementary Table 2a.** Associations between haplotypes in *IGF1* and breast cancer risk and mean IGF-I and IGFBP-3 levels.

| *IGF1* Haplotypes^a^ | Haplotype Frequency |  | Model | | |
| --- | --- | --- | --- | --- | --- |
|  |  |  | Codominant | Dominant | Recessive |
| hCCATC | 0.40 | OR (95% CI)^b^ | 1.00 | 1.00 | 1.00 |
|  |  | Alpha IGF-I | 422.41 | 420.34 | 428.77 |
|  |  | Alpha IGFBP-3 | 2469 | 2464 | 2464 |
|  |  |  |  |  |  |
| hCCATT | 0.24 | OR (95% CI)^b^ | 0.94 (0.79-1.12) | 0.88 (0.70-1.10) | 1.06 (0.74-1.52) |
|  |  | Beta IGF-I (*P*)^c^ | 2.60 (0.37) | 5.63 (0.14) | -4.51 (0.46) |
|  |  | Beta IGFBP-3 (*P*)^c^ | -15.21 (0.60) | -4.61 (0.90) | -36.08 (0.55) |
|  |  |  |  |  |  |
| hCCACC | 0.10 | OR (95% CI)^b^ | 1.07 (0.83-1.38) | 1.03 (0.77-1.39) | 1.59 (0.72-3.49) |
|  |  | Beta IGF-I (*P*)^c^ | 6.64 (0.12) | 8.31 (0.09) | 10.78 (0.44) |
|  |  | Beta IGFBP-3 (*P*)^c^ | -21.86 (0.61) | -23.41 (0.63) | 116.09 (0.40) |
|  |  |  |  |  |  |
| hCCACT | 0.08 | OR (95% CI)^b^ | 1.07 (0.82-1.39) | 1.05 (0.78-1.42) | 1.63 (0.68-3.94) |
|  |  | Beta IGF-I (*P*)^c^ | -3.18 (0.46) | -2.77 (0.57) | -21.55 (0.17) |
|  |  | Beta IGFBP-3 (*P*)^c^ | -8.60 (0.84) | -25.28 (0.60) | 262.29 (0.10) |
|  |  |  |  |  |  |
| hATGCC | 0.06 | OR (95% CI)^b^ | 0.86 (0.65-1.14) | 0.84 (0.62-1.14) | 0.80 (0.25-2.56) |
|  |  | Beta IGF-I (*P*)^c^ | **9.11 (0.04)** | **12.69 (0.01)** | -20.25 (0.28) |
|  |  | Beta IGFBP-3 (*P*)^c^ | -16.95 (0.70) | -15.37 (0.75) | 17.96 (0.92) |
|  |  |  |  |  |  |
| hATGTT | 0.05 | OR (95% CI)^b^ | 0.89 (0.65-1.22) | 0.89 (0.63-1.24) | 0.75 (0.20-2.85) |
|  |  | Beta IGF-I (*P*)^c^ | 0.03 (1.00) | 1.34 (0.81) | -18.18 (0.38) |
|  |  | Beta IGFBP-3 (*P*)^c^ | 39.17 (0.43) | 50.70 (0.34) | -26.82 (0.89) |
|  |  |  |  |  |  |
| hCTGCT | 0.02 | OR (95% CI)^b^ | 0.79 (0.48-1.32) | 0.79 (0.47-1.31) | - |
|  |  | Beta IGF-I (*P*)^c^ | 3.29 (0.69) | 3.77 (0.65) | - |
|  |  | Beta IGFBP-3 (*P*)^c^ | **-195.06 (0.02)** | **-195.64 (0.02)** | - |
|  |  |  |  |  |  |
| hCTGTT | 0.01 | OR (95% CI)^b^ | **0.40 (0.20-0.81)** | **0.40 (0.20-0.81)** | - |
|  |  | Beta IGF-I (*P*)^c^ | -2.98 (0.76) | -2.38 (0.81) | - |
|  |  | Beta IGFBP-3 (*P*)^c^ | **-286.69 (0.003)** | **-249.63 (0.01)** | - |
|  |  |  |  |  |  |
| hATGTC | 0.01 | OR (95% CI)^b^ | 0.80 (0.35-1.85) | 0.77 (0.33-1.78) | - |
|  |  | Beta IGF-I (*P*)^c^ | 11.82 (0.38) | 12.66 (0.34) | - |
|  |  | Beta IGFBP-3 (*P*)^c^ | -100.54 (0.44) | -99.42 (0.45) | - |
|  |  |  |  |  |  |

^a^ The order of SNPs in the haplotypes corresponds to their physical order: rs35765, rs35767, rs2162679, rs6220, rs6214.

^b^ OR = Odds ratio; CI = confidence interval; Reference group = hCTACC

^c^ Beta estimate for IGF-I and IGFBP-3 levels (in ng/mL) for a one unit change in haplotype dosage adjusted for age and center

**Supplementary Table 2b.** Associations between haplotypes in *IGF1*, limited to LD block at the 5’ of the gene, and breast cancer risk and mean IGF-I and IGFBP-3 levels.

| *IGF1* haplotypes^a^ | Haplotype Frequency |  | Model | | |
| --- | --- | --- | --- | --- | --- |
|  |  |  | Codominant | Dominant | Recessive |
|  |  |  |  |  |  |
| hCCA | 0.82 | OR (95% CI)^b^ | 1.00 | 1.00 | 1.00 |
|  |  | Alpha IGF-I | 425.61 | 424.57 | 428.32 |
|  |  | Alpha IGFBP-3 | 2471 | 2470 | 2470 |
|  |  |  |  |  |  |
| hATG | 0.13 | OR (95% CI)^b^ | 0.88 (0.74-1.06) | 0.87 (0.71-1.07) | 0.87 (0.46-1.63) |
|  |  | Beta IGF-I (*P*)^c^ | 4.15 (0.16) | **6.96 (0.04)** | -16.59 (0.11) |
|  |  | Beta IGFBP-3 (*P*)^c^ | 3.66 (0.90) | 10.50 (0.75) | -47.31 (0.64) |
|  |  |  |  |  |  |
| hCTG | 0.04 | OR (95% CI)^b^ | **0.69 (0.49-0.97)** | **0.68 (0.48-0.96)** | 0.95 (0.08-10.70) |
|  |  | Beta IGF-I (*P*)^c^ | 0.32 (0.95) | 1.36 (0.80) | -48.17 (0.23) |
|  |  | Beta IGFBP-3 (*P*)^c^ | **-227.44 (<0.0001)** | **-217.91 (<0.0001)** | **-1146.17 (0.004)** |
|  |  |  |  |  |  |

^a^ The order of SNPs in the haplotypes corresponds to their physical order: rs35765, rs35767, rs2162679.

^b^ OR = Odds ratio; CI = confidence interval; Reference group = hCCA

^c^ Beta estimate for IGF-I and IGFBP-3 levels (in ng/mL) for a one unit change in haplotype dosage adjusted for age and center

**Supplementary Table 2c.** Associations between haplotypes in *IGFBP1* and breast cancer risk and mean IGF-I and IGFBP-3 levels.

| *IGFBP1* Haplotypes^a^ | Haplotype Frequency |  | Model | | |
| --- | --- | --- | --- | --- | --- |
|  |  |  | Codominant | Dominant | Recessive |
|  |  |  |  |  |  |
| hGGCTAGT | 0.40 | OR (95% CI)^b^ | 1.00 | 1.00 | 1.00 |
|  |  | Alpha IGF-I | 427.00 | 426.99 | 426.42 |
|  |  | Alpha IGFBP-3 | 2490 | 2487 | 2484 |
|  |  |  |  |  |  |
| hAGCTAGT | 0.21 | OR (95% CI)^b^ | 0.89 (0.74-1.07) | 0.86 (0.69-1.08) | 1.07 (0.68-1.67) |
|  |  | Beta IGF-I (*P*)^c^ | -0.69 (0.81) | 0.20 (0.95) | -7.96 (0.29) |
|  |  | Beta IGFBP-3 (*P*)^c^ | -0.12 (1.00) | 2.90 (0.93) | -24.92 (0.74) |
|  |  |  |  |  |  |
| hGAACGGC | 0.17 | OR (95% CI)^b^ | 0.94 (0.77-1.16) | 0.97 (0.77-1.22) | 0.81 (0.45-1.45) |
|  |  | Beta IGF-I (*P*)^c^ | 0.95 (0.76) | 1.01 (0.76) | 3.36 (0.72) |
|  |  | Beta IGFBP-3 (*P*)^c^ | 14.60 (0.63) | 15.31 (0.65) | 46.41 (0.62) |
|  |  |  |  |  |  |
| hGACCGGC | 0.12 | OR (95% CI)^b^ | 0.93 (0.76-1.14) | 0.94 (0.75-1.18) | 0.84 (0.41-1.70) |
|  |  | Beta IGF-I (*P*)^c^ | 1.15 (0.73) | 0.53 (0.89) | 12.69 (0.27) |
|  |  | Beta IGFBP-3 (*P*)^c^ | -70.59 (0.04) | -75.16 (0.04) | -149.49 (0.19) |
|  |  |  |  |  |  |
| hAACCGAC | 0.04 | OR (95% CI)^b^ | 0.84 (0.61-1.17) | 0.86 (0.61-1.22) | 0.79 (0.15-4.07) |
|  |  | Beta IGF-I (*P*)^c^ | 0.44 (0.93) | 1.24 (0.82) | -6.25 (0.81) |
|  |  | Beta IGFBP-3 (*P*)^c^ | 55.19 (0.29) | 48.93 (0.37) | 384.62 (0.14) |
|  |  |  |  |  |  |
| hGAACGGT | 0.03 | OR (95% CI)^b^ | 0.86 (0.58-1.26) | 0.90 (0.60-1.35) | 0.40 (0.05-3.42) |
|  |  | Beta IGF-I (*P*)^c^ | -5.19 (0.40) | -4.81 (0.46) | -21.11 (0.46) |
|  |  | Beta IGFBP-3 (*P*)^c^ | 120.46 (0.05) | 136.66 (0.03) | 39.72 (0.89) |
|  |  |  |  |  |  |
| hAACCGGT | 0.02 | OR (95% CI)^b^ | 1.38 (0.87-2.19) | 1.38 (0.85-2.26) | 3.87 (0.35-42.75) |
|  |  | Beta IGF-I (*P*)^c^ | -0.14 (0.98) | -2.58 (0.73) | 74.09 (0.06) |
|  |  | Beta IGFBP-3 (*P*)^c^ | 13.41 (0.85) | 8.29 (0.91) | 213.79 (0.59) |
|  |  |  |  |  |  |

^a^ The order of SNPs in the haplotypes corresponds to their physical order: rs1995051, rs1065780, rs9658194, rs3828998, rs3793344, rs4988515, rs4619

^b^ OR = Odds ratio; CI = confidence interval; Reference group = hGGCTAGT

^c^ Beta estimate for IGF-I and IGFBP-3 levels (in ng/mL) for a one unit change in haplotype dosage adjusted for age and center

**Supplementary Table 2d.** Associations between haplotypes in *IGFBP3* and breast cancer risk and mean IGF-I and IGFBP-3 levels.

| IGFBP-3 Haplotypes^a^ | Haplotype Frequency |  | Model | | |
| --- | --- | --- | --- | --- | --- |
|  |  |  | Codominant | Dominant | Recessive |
| hGAGGCATA | 0.18 | OR (95% CI)^b^ | 1.00 | 1.00 | 1.00 |
|  |  | Alpha IGF-I | 429.13 | 431.66 | 427.30 |
|  |  | Alpha IGFBP-3 | 2441 | 2465 | 2470 |
|  |  |  |  |  |  |
| hGGTGCATA | 0.17 | OR (95% CI)^b^ | 0.99 (0.79-1.23) | 0.92 (0.72-1.18) | 1.15 (0.71-1.86) |
|  |  | Beta IGF-I (*P*)^c^ | 0.95 (0.80) | -1.98 (0.63) | **17.84 (0.03)** |
|  |  | Beta IGFBP-3 (*P*)^c^ | **111.34 (0.002)** | **103.78 (0.01)** | **200.56 (0.01)** |
|  |  |  |  |  |  |
| hGGTGCACA | 0.12 | OR (95% CI)^b^ | 1.06 (0.83-1.27) | 1.04 (0.80-1.35) | 1.02 (0.45-2.30) |
|  |  | Beta IGF-I (*P*)^c^ | -6.01 (0.14) | -8.14 (0.06) | -1.48 (0.91) |
|  |  | Beta IGFBP-3 (*P*)^c^ | **84.42 (0.03)** | 78.19 (0.06) | 82.22 (0.54) |
|  |  |  |  |  |  |
| hAGGCCATT | 0.09 | OR (95% CI)^b^ | 0.95 (0.72-1.24) | 0.84 (0.63-1.12) | **2.66 (1.10-6.42)** |
|  |  | Beta IGF-I (*P*)^c^ | -2.53 (0.56) | -5.62 (0.22) | 21.64 (0.16) |
|  |  | Beta IGFBP-3 (*P*)^c^ | **-92.12 (0.03)** | **-124.44 (0.01)** | -87.82 (0.56) |
|  |  |  |  |  |  |
| hGGTGCATT | 0.09 | OR (95% CI)^b^ | 1.08 (0.81-1.42) | 1.02 (0.76-1.37) | 1.86 (0.72-4.83) |
|  |  | Beta IGF-I (*P*)^c^ | -5.62 (0.22) | -7.63 (0.11) | 5.96 (0.72) |
|  |  | Beta IGFBP-3 (*P*)^c^ | **164.72 (0.0002)** | **159.04 (0.0008)** | 321.67 (0.05) |
|  |  |  |  |  |  |
| hAGGGCGTA | 0.09 | OR (95% CI)^b^ | 0.92 (0.70-1.21) | 0.85 (0.64-1.12) | 2.00 (0.64-6.26) |
|  |  | Beta IGF-I (*P*)^c^ | -4.69 (0.29) | -5.88 (0.19) | -5.84 (0.77) |
|  |  | Beta IGFBP-3 (*P*)^c^ | -31.86 (0.46) | -46.98 (0.29) | -125.91 (0.52) |
|  |  |  |  |  |  |
| hAGGCCATA | 0.08 | OR (95% CI)^b^ | 1.01 (0.76-1.34) | 0.93 (0.69-1.27) | 1.71 (0.66-4.44) |
|  |  | Beta IGF-I (*P*)^c^ | -0.06 (0.99) | -0.52 (0.92) | -1.74 (0.92) |
|  |  | Beta IGFBP-3 (*P*)^c^ | -14.12 (0.76) | -26.29 (0.60) | -121.82 (0.46) |
|  |  |  |  |  |  |
| hGGTGCGTA | 0.07 | OR (95% CI)^b^ | 0.90 (0.66-1.23) | 0.83 (0.60-1.15) | 1.45 (0.39-5.46) |
|  |  | Beta IGF-I (*P*)^c^ | 1.91 (0.71) | 1.33 (0.80) | -24.37 (0.29) |
|  |  | Beta IGFBP-3 (*P*)^c^ | 73.41 (0.15) | 71.81 (0.17) | -164.46 (0.47) |
|  |  |  |  |  |  |
| hGGTGCGTT | 0.02 | OR (95% CI)^b^ | 0.68 (0.38-1.22) | 0.66 (0.37-1.19) | - |
|  |  | Beta IGF-I (*P*)^c^ | -10.96 (0.24) | -12.32 (0.18) | - |
|  |  | Beta IGFBP-3 (*P*)^c^ | **210.34 (0.02)** | **194.49 (0.03)** | - |
|  |  |  |  |  |  |
| hAGGGCATA | 0.02 | OR (95% CI)^b^ | **0.52 (0.29-0.95)** | **0.51 (0.27-0.94)** | - |
|  |  | Beta IGF-I (*P*)^c^ | -10.08 (0.24) | -13.85 (0.13) | - |
|  |  | Beta IGFBP-3 (*P*)^c^ | -145.64 (0.08) | -140.18 (0.11) | - |
|  |  |  |  |  |  |

^a^ The order of SNPs in the haplotypes corresponds to their physical order: rs2132571, rs2132572, rs2854744, rs2471551, P0453, rs2453839, P0448, rs6670.

^b^ OR = Odds ratio; CI = confidence interval; Reference group = hATACGGAG

^c^ Beta estimate for IGF-I and IGFBP-3 levels (in ng/mL) for a one unit change in haplotype dosage adjusted for age and center

**Supplementary Table 2e.** Associations between haplotypes in *IGFBP3*, limited to LD block at the 5’ of the gene, and breast cancer risk and mean IGF-I and IGFBP-3 levels.

| *IGFBP3* haplotypes^a^ | Haplotype Frequency |  | Model | | |
| --- | --- | --- | --- | --- | --- |
|  |  |  | Codominant | Dominant | Recessive |
|  |  |  |  |  |  |
| hGGT | 0.49 | OR (95% CI)^b^ | 1.00 | 1.00 | 1.00 |
|  |  | Alpha IGF-I | 424.03 | 426.80 | 425.51 |
|  |  | Alpha IGFBP-3 | 2660 | 2644 | 2500 |
|  |  |  |  |  |  |
| hAGG | 0.31 | OR (95% CI)^b^ | 0.94 (0.82-1.09) | 0.86 (0.72-1.03) | 1.15 (0.86-1.55) |
|  |  | Beta IGF-I (*P*)^c^ | 1.86 (0.43) | 0.29 (0.92) | 4.91 (0.33) |
|  |  | Beta IGFBP-3 (*P*)^c^ | **-162.39 (<0.0001)** | **-168.84 (<0.0001)** | **-231.16 (<0.0001)** |
|  |  |  |  |  |  |
| hGAG | 0.20 | OR (95% CI)^b^ | 1.00 (0.85-1.17) | 0.93 (0.78-1.12) | 1.33 (0.90-1.99) |
|  |  | Beta IGF-I (*P*)^c^ | 2.96 (0.27) | -0.41 (0.90) | 19.56 (0.004) |
|  |  | Beta IGFBP-3 (*P*)^c^ | **-115.59 (<0.0001)** | **-115.89 (0.0002)** | -69.21 (0.30) |
|  |  |  |  |  |  |

^a^ The order of SNPs in the haplotypes corresponds to their physical order: rs2132571, rs2132572, rs2854744.

^b^ OR = Odds ratio; CI = confidence interval; Reference group = hGGT

^c^ Beta estimate for IGF-I and IGFBP-3 levels (in ng/mL) for a one unit change in haplotype dosage adjusted for age and center

**Supplementary Table 2f.** Associations between haplotypes in *IGFALS* and breast cancer risk and mean IGF-I and IGFBP-3 levels.

| *IGFALS* Haplotypes^a^ | Haplotype Frequency |  | Model | | |
| --- | --- | --- | --- | --- | --- |
|  |  |  | Codominant | Dominant | Recessive |
|  |  |  |  |  |  |
| hCTG | 0.73 | OR (95% CI)^b^ | 1.00 | 1.00 | 1.00 |
|  |  | Alpha IGF-I | 430.59 | 429.30 | 431.29 |
|  |  | Alpha IGFBP-3 | 2506 | 2501 | 2482 |
|  |  |  |  |  |  |
| hCCG | 0.17 | OR (95% CI)^b^ | 1.06 (0.90-1.25) | 1.08 (0.89-1.29) | 0.98 (0.58-1.67) |
|  |  | Beta IGF-I (*P*)^c^ | -5.21 (0.06) | -3.60 (0.25) | **-24.88 (0.005)** |
|  |  | Beta IGFBP-3 (*P*)^c^ | **-**51.87 (0.06) | -54.21 (0.08) | **-102.73 (0.24)** |
|  |  |  |  |  |  |
| hTTG | 0.10 | OR (95% CI)^b^ | 1.15 (0.93-1.41) | 1.16 (0.93-1.44) | 1.14 (0.41-3.14) |
|  |  | Beta IGF-I (*P*)^c^ | 0.78 (0.83) | 2.19 (0.56) | -18.58 (0.29) |
|  |  | Beta IGFBP-3 (*P*)^c^ | -30.85 (0.38) | -35.56 (0.33) | 64.99 (0.70) |
|  |  |  |  |  |  |

^a^ The order of SNPs in the haplotypes corresponds to their physical order: rs3751893, rs17559, rs2230053.

^b^ OR = Odds ratio; CI = confidence interval; Reference group = hGCT

^c^ Beta estimate for IGF-I and IGFBP-3 levels (in ng/mL) for a one unit change in haplotype dosage adjusted for age and center
